# Frizzled-2: A potential novel target for molecular pancreatic cancer therapy

**DOI:** 10.3892/ol.2013.1681

**Published:** 2013-11-12

**Authors:** MINORU TOMIZAWA, FUMINOBU SHINOZAKI, TAKAO SUGIYAMA, SHIGENORI YAMAMOTO, MAKOTO SUEISHI, TAKANOBU YOSHIDA

**Affiliations:** 1 Department of Gastroenterology, National Hospital Organization, Shimoshizu Hospital, Yotsukaido, Chiba 284-0003, Japan; 2 Department of Radiology, National Hospital Organization, Shimoshizu Hospital, Yotsukaido, Chiba 284-0003, Japan; 3 Department of Rheumatology, National Hospital Organization, Shimoshizu Hospital, Yotsukaido, Chiba 284-0003, Japan; 4 Department of Pediatrics, National Hospital Organization, Shimoshizu Hospital, Yotsukaido, Chiba 284-0003, Japan; 5 Department of Internal Medicine, National Hospital Organization, Shimoshizu Hospital, Yotsukaido, Chiba 284-0003, Japan

**Keywords:** frizzled genes, β-catenin, quantitative PCR, MTS assay

## Abstract

In the present study, pancreatic cancer cell proliferation was analyzed following the suppression of frizzled (Fz)2 expression. Reverse transcription polymerase chain reaction (PCR) was performed using RNA isolated from pancreatic cancer cell lines, PANC-1, NOR-P1, PK-45H, PK-1, PK-59, MIA-Paca2 and KP4. A surgical specimen of pancreatic cancer was immunostained with antibodies specific to Fz2. Cell proliferation assays were performed with MIA-Paca2 cells transfected with small interfering RNA (siRNA) or short hairpin RNA (shRNA) of Fz2. Fz2 was found to be expressed in all pancreatic cancer cell lines, with the exception of NOR-P1. Immunostaining revealed that Fz2 was not expressed in normal pancreatic tissues, while it was expressed in pancreatic cancer cells. The expression levels of cyclin D1 were analyzed by quantitative PCR. The proliferation and expression of cyclin D1 were suppressed with the siRNA and shRNA of Fz2 in the MIA-Paca2 cells. Therefore, Fz2 is a potential target for the molecular therapy of pancreatic cancer.

## Introduction

Pancreatic cancer is the fourth leading cause of cancer-related mortality (1). Furthermore, pancreatic cancer has a poor prognosis, with a 5-year survival rate of 6% (1), in part due to its ability to metastasize at an early stage (2). Non-cohesive pancreatic cancer is caused by the loss of expression of certain proteins, including E-cadherin, which results in cancer cell dissemination and subsequently, a poor prognosis (3,4). Consequently, only 10% of patients with pancreatic cancer are treated with curative therapy, whilst 90% are treated conservatively (5). Therefore, there is a requirement for the development of novel treatments for pancreatic cancer.

Small-molecule receptor tyrosine kinase inhibitors are currently under investigation as anticancer agents (6). Insulin-like growth factor-I receptor inhibitors are particularly promising, as they inhibit the dissemination of pancreatic cancer cells (7,8). One of the disadvantages of these inhibitors, however, is the observed co-inhibition of the insulin receptor (9).

The Wnt signaling pathway is pivotal for cell growth and differentiation. There are 3 signaling branches of the Wnt pathway, the canonical, planar-cell polarity and Wnt-Ca^2+^ pathways (10), with the former being the best characterized. During the canonical Wnt pathway, β-catenin is first phosphorylated and degraded in normal cells, following which, it enters the nucleus where it increases the expression of target genes, including cyclin D1, a protein involved in cell proliferation (11). Frizzled (Fz) is a receptor of Wnt ligand. Upon binding of Wnt ligand to Fz, the Wnt pathway is activated. In total, 10 members of the Fz family of genes have been identified based on structural and functional homology studies (12). Fz genes have been previously implicated in carcinogenesis and embryogenesis and their expression is upregulated in gastric cancer and hepatoma cells (13,14). We investigated the possibility of Fz2 as a potential target of molecular therapy for pancreatic cancer.

## Materials and methods

### Cell culture

Pancreatic cancer cell lines, PANC-1, MIA-Paca2, NOR-P1, PK-45H, PK-1, PK-59 and KP4, were purchased from Cell Bank, RIKEN BioResource Center (Tsukuba, Japan). MIA-Paca2 was cultured in Dulbecco’s modified Eagle’s medium (DMEM), KP4 was cultured in DMEM:F12 and the remaining lines were cultured in Roswell Park Memorial Institute medium-1640. Media were purchased from Sigma-Aldrich (St. Louis, MO, USA) and supplemented with 10% fetal bovine serum (FBS; Life Technologies, Carlsbad, CA, USA). The cell lines were cultured with 5% carbon dioxide at 37°C in a humidified chamber.

### Reverse transcription (RT) and quantitative polymerase chain reaction (PCR)

The cells were spread in 6-well plates (Asahi Glass Co., Ltd., Tokyo, Japan) and cultured. When the cells had reached 80% confluency, they were further cultured for 48 h after transfection. Total RNA (5 μg), isolated with Isogen (Nippon Gene Co., Ltd., Tokyo, Japan), was used to generate cDNA with Super Script III and oligo dT primers, according to the manufacturer’s instructions (Life Technologies). Human whole pancreas RNA was purchased from Takara and used as a positive control (Takara Bio, Inc., Shiga, Japan). The PCR primers, annealing temperatures, reaction cycle numbers and product sizes were as follows: Fz1 (GenBank accession no. NM_003505) forward (F), 5′-AATGACAAGTTCGCC GAGGAC-3′ and reverse (R), 5′-GCCAGGTGAAAATACTGT GAGTTGG-3′ (59°C for 30 cycles; 206 bp); Fz2 (NM_001466) F, 5′-CAAGGTGCCATCCTATCTCAGC-3′ and R, 5′-GTA GCAGCCCGACAGAAAAATG-3′ (59°C for 30 cycles; 247 bp); Fz3 (NM_017412) F, 5′-AGAGAAGAACTGTCATTT GCTCGC-3′ and R, 5′-TCCTTGTGTCACTGTGGAAGCC-3′ (53°C for 30 cycles; 255 bp); Fz4 (NM_012193) F, 5′-CAAGTG ATTCTCCTGCCACAGC-3′ and R, 5′-CAACTCTCTCCA GTGTCCTCCATC-3′ (57°C for 30 cycles; 270 bp); Fz5 (NM_003468) F, 5′-CCCTCATCCCCTAAGAGAGAC AAAG-3′ and R, 5′-GCTGGCTGTGAAGAAGTTGCTG-3′ (55°C for 30 cycles; 230 bp); Fz6 (NM_003506) F, 5′-AGCAGC ATCCATCTCCAGACTCTC-3′ and R, 5′-CTGAATGACAAC CACCTCCCTG-3′, (57°C for 30 cycles, 251 bp); Fz7 (NM_003507) F, 5′-AGACTTAGCCACAGCAGCAAGG-3′ and R, 5′-CGCCGTTATCATCATCTTCCTG-3′ (58°C for 30 cycles; 287 bp); Fz8 (NM_031866) F, 5′-ATCCAAAGCAGA TGCCATTGTC-3′ and R, 5′-AACACTGTGAAGGGGTGG GAAC-3′ (59°C for 30 cycles; 137 bp); Fz9 (BC_026333) F, 5′-TCTTTGGAGAACCCCACACACC-3′ and R, 5′-TGC TCACTTGCCTGACCTTGAC-3′ (60°C for 30 cycles; 148 bp); Fz10 (NM_007197) F, 5′-AAACGCTGGACTGCCTGATG-3′ and R, 5′-GCTTTTTTGTAAATCCCACCGC-3′ (58°C for 30 cycles; 217 bp); and GAPDH (NM_002046) F, 5′-ACCTGA CCTGCCGTCTAGAA-3′ and R, 5′-TCCACCACCCTGTTG CTGTA-3′ (63°C for 30 cycles; 246 bp). PCR was performed using Taq DNA polymerase (Life Technologies) and products were subjected to analysis by gel electrophoresis in 2% agarose in 1X TAE (40 mM Tris-acetate/1 mM EDTA). Quantitative PCR was performed using Fast SYBR Green Master Mix (Life Technologies) and analyzed with the MiniOpticon Detection System (Bio-Rad, Hercules, CA, USA). The primer pairs for quantitative PCR and the resultant product sizes were as follows: Fz2 (NM_001466) F, 5′-TCCTCAAGGTGCCAT CCTATCTC-3′ and R, 5′-TGGTGACAGTGAAGAAGGTGG AAG-3′ (183 bp); cyclin D1 (NM_053056) F, 5′-AGAGGCGGA GGAGAACAAACAG-3′ and R, 5′-AGGCGGTAGTAGGAC AGGAAGTTG-3′ (180 bp); and ribosomal protein L19 (RPL19; BC095445) F, 5′-CGAATGCCAGAGAAGGTCAC-3′ and R, 5′-CCATGAGAATCCGCTTGTTT-3′ (157 bp). Quantitative PCR was performed for 40 cycles of 5 sec for denaturation and 5 sec for annealing/extension. GAPDH and RPL19 were used as internal controls.

### Immunostaining and microscopy

Serial sections of human pancreatic cancer tissue (62-year-old male with adenocarcinoma; BioChain Institute, Inc., Newark, CA, USA) were deparaffinized, autoclaved and incubated first with hydrogen peroxide, followed by a 30-min incubation with 2% normal goat serum in phosphate-buffered saline (PBS; washing buffer). Following overnight incubation with a rabbit polyclonal anti-Fz2 antibody (1:5,000; Sigma-Aldrich), the specimens were rinsed with PBS and subsequently incubated with horseradish peroxidase-labeled anti-rabbit antibody (1:500; GE Healthcare, Amersham, UK) for 2 h. Next, diaminobenzidine (Dako, Carpinteria, CA, USA) was applied to the tissue sections as a chromogen, and the nuclei were stained with hematoxylin (Muto Pure Chemicals Co., Ltd., Tokyo, Japan) for 15 sec. The specimens were observed and images were captured under an AX80 microscope (Olympus Corp., Tokyo, Japan).

### Cell proliferation analysis

The MIA-Paca2 cells were trypsinized, harvested and spread onto 96-well flat-bottom plates (Asahi Techno Glass) at a density of 1,000 cells per well. Next, the cells were incubated for 24 h in DMEM supplemented with 10% FBS. Following culture, the cells were transfected with the short interference RNA (siRNA) of Fz2 (siRNA-Fz2) or the short hairpin RNA (shRNA) of Fz2 (shRNA-Fz2) for 72 h. Cell cultures were then used in 3-(4,5-dimethylthiazol-2-yl)-5-(3-carboxymethoxyphenyl)-2-(4-sulfophenyl)-2H-tetrazolium inner salt (MTS) assays, according to the manufacturer’s instructions (Promega Corporation, Madison, MI, USA). MTS is bio-reduced by cells into a colored formazan product that reduces absorbance at 490 nm. Absorbance was analyzed at a wavelength of 490 nm with an iMark Microplate Absorbance Reader (Bio-Rad).

### siRNA and shRNA transfection

Transfection of the cells with siRNA-Fz2 (Life Technologies) was carried out using Lipofectamine 2000 and Opti-MEM (both Life Technologies), according to the manufacturer’s instructions. Briefly, siRNA and Lipofectamine 2000 were separately diluted in Opti-MEM at room temperature for 5 min. The diluted siRNA and Lipofectamine 2000 were then incubated together for an additional 20 min at room temperature to facilitate complex formation. Next, culture medium was aspirated from dishes or wells containing cells and the complexes were added to cultured cells. shRNA-Fz2 (OriGene Technologies Inc., Rockville, MD, USA) was transfected into cells using Lipofectamine LTX (Life Technologies), according to the manufacturer’s instructions. Briefly, shRNA was incubated with PLUS reagent for 5 min, following which, LTX reagent was added. A 30-min incubation at room temperature ensued and the complex was subsequently applied to the cell culture medium.

### Statistical analysis

Cell proliferation and quantitative PCR data were analyzed by a one-way analysis of variance. The statistical analysis was performed using JMP 5.0 software (SAS Institute Inc., Cary, NC, USA) and P<0.05 was considered to indicate a statistically significant difference.

## Results

### Fz gene expression levels in pancreatic tissue

**Figure 1:**
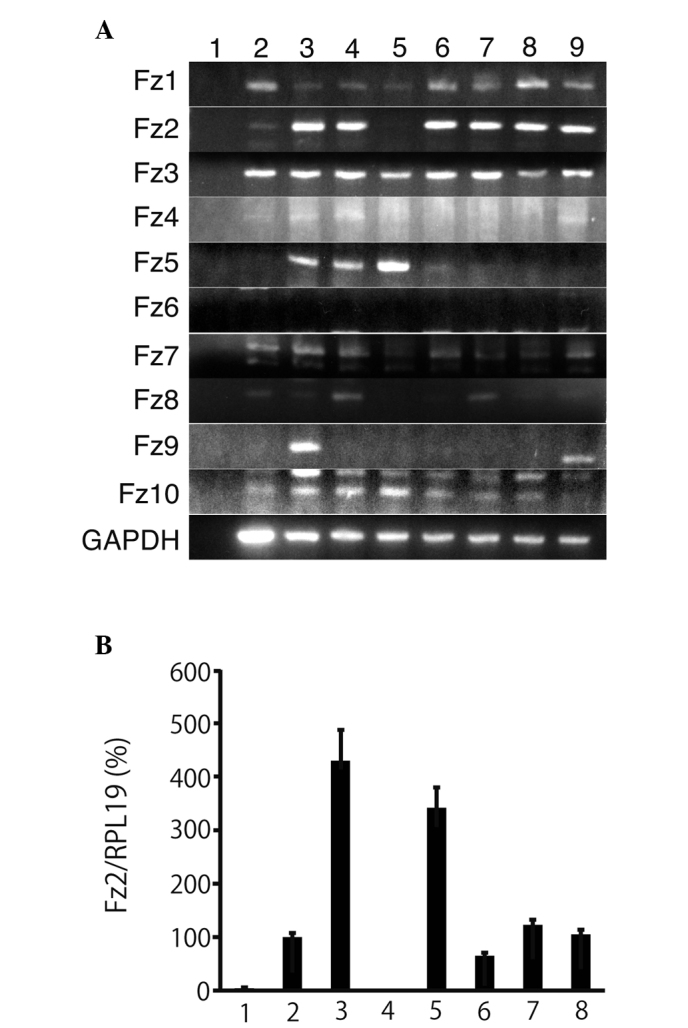
Expression of Fz genes in pancreatic cancer cell lines. (A) Expression of Fz genes was analyzed in pancreatic cancer cell lines by RT-PCR. Lane 1, H_2_O; 2, normal pancreatic tissue; 3, PANC-1; 4, MIA-Paca2; 5, NOR-P1; 6, PK-45H; 7, PK-59; 8, PK-1; and 9, KP4. Fz2/RLP19 was calculated as the expression level of Fz2. All experiments were performed in triplicate. (B) Expression levels of Fz2 were analyzed in pancreatic cancer cell lines by quantitative PCR. Lane 1, normal pancreas; 2, MIA-Paca2; 3, PANC-1; 4, NOR-P1; 5, PK-45H; 6, PK-59; 7, PK-1; and 8, KP4. Fz, frizzled; RT-PCR, reverse transcription polymerase chain reation; RPL19, ribosomal protein L19.

The expression levels of the Fz genes were analyzed by RT-PCR in the normal pancreatic tissue and the pancreatic cancer cell lines (PANC-1, MIA-Paca2, NOR-P1, PK-45H, PK-1, PK-59 and KP4; Fig. 1A). Fz1, Fz3, Fz7 and Fz10 were expressed not only in the pancreatic cancer cells, but also in the normal pancreatic tissues, while Fz6 was not expressed in either of the two cell types. Fz4 was only weakly expressed in the pancreatic cancer cell lines. In addition, Fz5 and Fz9 were not expressed in the normal pancreatic tissues, but were expressed in 3 and 1 of the pancreatic cancer cell lines, respectively. Fz8 was expressed only in the normal pancreatic tissues, while Fz2 was weakly expressed in the normal pancreatic tissues and expressed in all other pancreatic cancer cell lines examined, with the exception of NOR-P1. Relative to its expression in MIA-Paca2, the expression levels of Fz2 in the normal pancreatic tissue was 4.1±1.8% (Fig. 1B). The expression of Fz2 was 1.3±0.7% of NOR-P1. These expression results were consistent with the RT-PCR results, and Fz2 was consequently used for further analysis.

### Immunostaining

**Figure 2:**
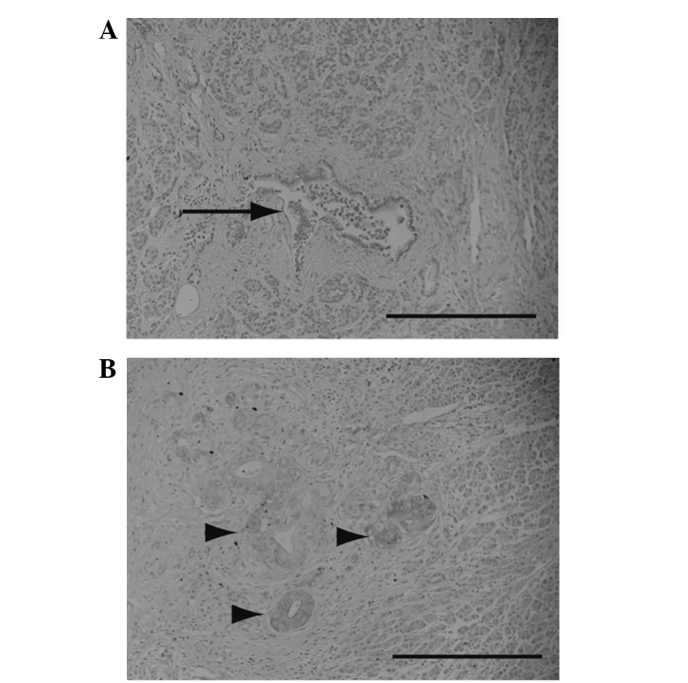
Immunostaining. Surgical specimens of (A) normal pancreatic duct tissues were compared with (B) specimens obtained from a patient with pancreatic cancer and immunostained with an antibody against Fz2. Arrow indicates the normal pancreatic duct and arrowheads indicate cancer (magnification, ×200; scale bar, 100 μm). Fz2, frizzled-2.

Immunostaining was performed to determine the expression of Fz2 at the protein level in the normal pancreatic and pancreatic cancer tissues (Fig. 2). Fz2 was not expressed in the glands or normal pancreatic duct tissues (Fig. 2A). By contrast, Fz2 was expressed in the pancreatic cancer cells (Fig. 2B).

### Inhibition of Fz2 expression

**Figure 3:**
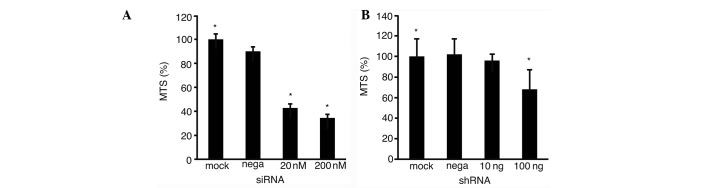
Cell proliferation analysis. Cell proliferation was analyzed in MIA-Paca2 cells by MTS assays 72 h after the transfection of cells with (A) the siRNA or (B) shRNA of Fz2. ^*^P<0.05 (one-way analysis of variance). Experiments were performed in triplicate. siRNA, small interfering RNA; shRNA, short hairpin RNA; Fz2, frizzled-2; mock, mock transfected; nega, transfected with negative control; MTS, 3-(4,5-dimethylthiazol-2-yl)-5-(3-carboxymethoxyphenyl)-2-(4-sulfophenyl)-2H-tetrazolium inner salt.

**Figure 4:**
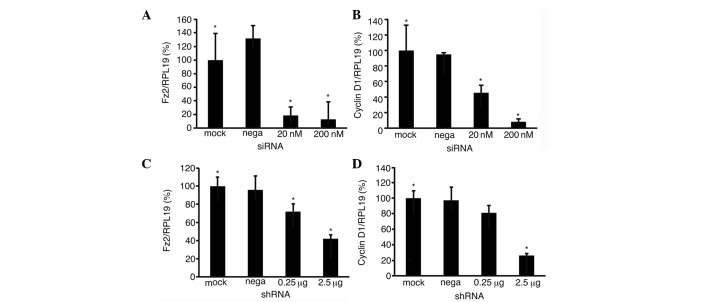
Quantitative PCR. Expression levels of Fz2 and cyclin D1 were analyzed in MIA-Paca2 cells transfected with (A and B) the siRNA or (C and D) shRNA of Fz2. The groups were as follows: Mock, mock transfected; nega, transfected with negative control; 20 nM, transfected with 20 nM siRNA; 200 nM, transfected with 200 nM siRNA; 0.25 μg, transfected with 0.25 μg shRNA; and 2.5 μg, transfected with 25 μg shRNA. ^*^P<0.05 (one-way analysis of variance). Experiments were performed in triplicate. PCR, polymerase chain reaction; Fz2, frizzled-2; siRNA, small interfering RNA; shRNA, short hairpin RNA; RPL19, ribosomal protein L19.

These results prompted the study of the suppression of pancreatic cancer cell proliferation following the inhibition of Fz2 expression. MIA-Paca2 cells were cultured with siRNA- or shRNA-Fz2 and subjected to MTS assays. Relative to the mock transfection, the proliferation of the MIA-Paca2 cells was suppressed to 34.5±2.8% (P<0.05; Fig. 3A) following transfection with 200 nM siRNA-Fz2, and to 68.0±19.0% (P<0.05) following transfection with 100 ng/well shRNA-Fz2 (Fig. 3B). The expression levels of Fz2 were analyzed by quantitative PCR to confirm suppression with siRNA- or shRNA-Fz2 (Fig. 4). Fz2 expression was suppressed to 12.9±0.9% (P<0.05) following transfection with 200 nM siRNA-Fz2 (Fig. 4A), and to 42.0±4.1% (P<0.05) following transfection with 2.5 μg/well shRNA-Fz2 (Fig. 4C).

### Cyclin D1 expression

Finally, the expression of cyclin D1 was analyzed to investigate the mechanism of the observed suppression of cell proliferation in the MIA-Paca2 cells. Fz2 expression levels were suppressed to 8.2±3.7% (P<0.05) following transfection with 200 nM siRNA-Fz2 (Fig. 4B), and to 26.2±2.6% (P<0.05) following transfection with 2.5 μg/well shRNA-Fz2 (Fig. 4D).

## Discussion

Sagara *et al*(1998) reported that Fz2 was not expressed in the normal pancreas (15). Although the RT-PCR results of the current study revealed a weak band of Fz2 expression in the normal pancreatic tissue, the expression was significantly low. The discrepancy between the RT-PCR and quantitative PCR results may be attributed to the saturation of PCR products in the former method. The results of the quantitative PCR were consistent with that of previous studies. In pancreatic cancer patients, Fz2 is upregulated in 8 cases out of 15 (16). In the present study, relative to normal pancreatic tissues, Fz2 was overexpressed in 6 out of the 7 pancreatic cancer cell lines. The results of the quantitative PCR indicate, therefore, that Fz2 is upregulated in pancreatic cancer cell lines more often than in pancreatic cancer tissues. We hypothesize that the upregulation of Fz2 is necessary for cells to proliferate *in vitro*. Notably, β-catenin protein levels have been shown to be upregulated in patients whose cancerous pancreatic tissue showed increased expression levels of Fz2 (16), even though the gene encoding β-catenin was only mutated in 13.3% (2/15) of the patients (16). Using a global genomic analysis, a previous study found that 12 pathways were altered in 24 pancreatic cancer patients, to varying degrees (17). Alteration of the Wnt pathway is not specific to pancreatic cancer, but also occurs in colorectal cancer and hepatocellular carcinoma (18). Fz2 was has been found to be upregulated in 61.5% (8/13) of pancreatic cancer patients in the absence of the β-catenin mutation (16). APC is not mutated and β-catenin is not upregulated in MIA-Paca2 cells, a representative cell line of pancreatic cancer (19). In the present study, cellular transfection with siRNA- and shRNA-Fz2 suppressed the proliferation of the MIA-Paca2 cells, a result that we now expect to obtain in patient pancreatic cancer tissues containing normal APC and β-catenin.

Cyclin D1 is upregulated in pancreatic cancer tissues compared with surrounding normal tissues (20). Moreover, antisense oligonucleotides specific to cyclin D1 inhibit the growth of pancreatic cancer *in vivo*(21). Together, these results indicate that pancreatic cancer cell proliferation is suppressed with the inhibition of cyclin D1 expression. In cells of hepatocellular carcinoma, proliferation is suppressed with siRNA-Fz9 and is associated with the decreased expression of cyclin D1 (14). Similarly, in the present study, pancreatic cancer cell proliferation and the expression of cyclin D1 were suppressed following siRNA- and shRNA-Fz2 treatment, the latter as revealed by quantitative PCR analysis. The results are, therefore, consistent with previous observations. The main disadvantages of using antisense RNA in cancer treatment are that its half-life is short and its delivery is difficult (22). In contrast to cyclin D1, a cytoplasmic protein, Fz2, is a receptor. A novel molecular therapy for pancreatic cancer may, therefore, be developed using monoclonal antibodies and small molecule receptor inhibitors specific to Fz2. Future studies are likely to analyze the signaling pathway between Fz2 and cyclin D1 in pancreatic cells and tissues.
